# Antibacterial and Antibiofilm Potential of Ethanolic Extracts of *Duguetia vallicola* (Annonaceae) against in-Hospital Isolates of *Pseudomonas aeruginosa*

**DOI:** 10.3390/plants13101412

**Published:** 2024-05-18

**Authors:** Orfa Inés Contreras-Martínez, Daniela Sierra-Quiroz, Alberto Angulo-Ortíz

**Affiliations:** 1 Biology Department, Faculty of Basic Sciences, University of Córdoba, Montería 230002, Colombia; oicontreras@correo.unicordoba.edu.co (O.I.C.-M.); dsierraquiroz17@correo.unicordoba.edu.co (D.S.-Q.); 2 Chemistry Department, Faculty of Basic Sciences, University of Córdoba, Montería 230002, Colombia

**Keywords:** ethanolic extracts, *Duguetia vallicola*, antibacterial activity, antibiofilm, *Pseudomonas aeruginosa*

## Abstract

*Pseudomonas aeruginosa* is an opportunistic pathogen that is especially dominant in people with cystic fibrosis; the drug resistance expressed by this pathogen and its capacity for adaptation poses a significant challenge to its treatment and control, thereby increasing morbidity and mortality rates globally. In this sense, the search for new treatment alternatives is imminent today, with products of plant origin being an excellent alternative for use. The objective of this research was to evaluate the antibacterial and antibiofilm potential and to explore the possible effect of ethanolic extracts from the wood and bark of *Duguetia vallicola* on the cell membrane. Microdilution assays showed the inhibition of bacterial growth by more than 50%, with the lowest concentration (62.5 μg/mL) of both extracts evaluated. Furthermore, we report the ability of both extracts to inhibit mature biofilms, with inhibition percentages between 48.4% and 93.7%. Intracellular material leakage experiments (260/280 nm), extracellular pH measurements, and fluorescence microscopy with acridine orange (AO) and ethidium bromide (EB) showed cell membrane damage. This indicates that the antibacterial action of ethanolic extracts of *D. vallicola* is associated with damage to the integrity of the cell membrane and consequent death of these pathogens. These results serve as a reference for future studies in establishing the mechanisms of action of these extracts.

## 1. Introduction

*Pseudomonas aeruginosa* is a Gram-negative pathogen that causes illness and death in people with immunosuppressive and chronic conditions [1,2]. It is dominant in people with cystic fibrosis [3,4,5], burns, or those who have acquired this pathogen at the surgical site, causing coinfections and generalized sepsis [6]. Its high intrinsic resistance to antiseptics and antibiotics [7,8] makes it a pathogen of great clinical relevance due to the high rates of morbidity and mortality, especially in immunocompromised people [9]. According to the World Health Organization (WHO), these pathogens represent between 7% and 12% of isolates in healthcare-associated infections (HAIs) [10]. The pathogenicity, adaptability, and flexibility of *P. aeruginosa* are due to the expression of various virulence factors [1,3,7], highlighting the production of powerful biofilms [4,11] that facilitate their multidrug resistance and evasion of the immune system [12]. Thus, *P. aeruginosa* infections represent a serious public health problem at a global level [13]. Therefore, the search for therapeutic alternatives that help mitigate the problem generated by this pathogen is important today.

In this context, compounds of natural origin and their synthetic derivatives are an excellent alternative for use, especially those obtained from plants, since these are an invaluable source of metabolites with recognized medicinal properties [14,15]. Compounds obtained from members of the Annonaceae family are well known for their outstanding and important biological activity [16,17,18,19,20,21]. The genus *Duguetia* is one of the most abundant in this family, from which various compounds have been obtained, including terpenes, lignans, flavonoids, and aromatic compounds, and it has mainly been documented with a high content of alkaloids [22]. Alkaloids have been widely reported for their important biological activities, such as their antitumor [23,24], antimicrobial [25,26,27,28], trypanocidal, leishmanicidal [23], antiplasmodial [17], antiviral [29], antioxidant, anti-inflammatory and antinociceptive [30,31,32,33] properties, evidencing the variety of secondary metabolites, especially alkaloids, and their outstanding activity. 

In particular, alkaloids such as N-methylaurotetanine and its O-methylated and O-acetylated derivatives have been isolated and characterized from *Duguetia vallicola*, which have been documented to possess antioxidant activity [30]. Glaziovine, known for its neuropharmacological properties [34], and isoquinoline alkaloids (duguevalline, cleistopholine, O-methylmoschatoline, (-)-oliveroline and (-)-oliveridine) exhibit antiplasmodial activity against *Plasmodium falciparum* [17]. However, there are no reports on the antibacterial potential of *D. vallicola* extracts against clinical isolates of *P. aeruginosa*. We hypothesize that its bark and wood extracts have an antibacterial effect against *P. aeruginosa*. This research aimed to evaluate the antibacterial and antibiofilm potential of ethanolic extracts of *D. vallicola* wood and bark, as well as to explore its mechanism of action, targeting the cell membrane. The results of this research contribute to the search for molecules and compounds of plant origin with antimicrobial potential against *P. aeruginosa*.

## 2. Results

### 2.1. Identification of Alkaloids in the Wood Extract

**Figure 1:**
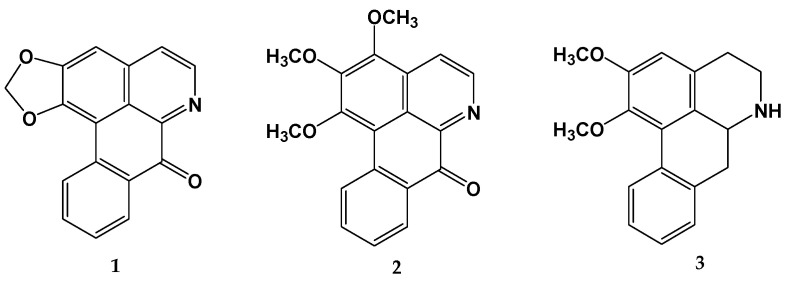
Structures of liriodenine (**1**), O-methylmoschatoline (**2**), and nornuciferine (**3**) isolated from the *Duguetia vallicola* wood extract.

Two alkaloids with an oxoaporphin nucleus, known as liriodenine (**1**) and O-methylmoschatoline (**2**), were obtained from the *D. vallicola* wood extract, along with an aporphinic alkaloid known as nornuciferine (**3**). Alkaloids **1** and **3** are reported for the first time in *D. vallicola*. Their structures (Figure 1) were established through a comparison of their physical and spectral data with those published in the literature. The ^1^H-NMR, ^13^C-NMR, HMQC, and HMBC data for compounds **1**, **2**, and **3** are presented in Tables S1–S3, respectively. The ^1^H-NMR spectra are presented in Figures S1–S5.

### 2.2. Antibacterial Susceptibility Testing

**Figure 2:**
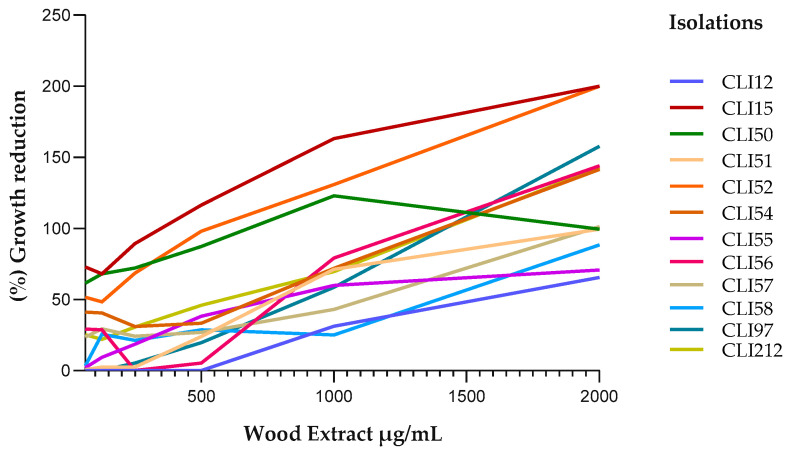
Reduction in the growth of *P. aeruginosa* isolates exposed to the ethanolic wood extract. A positive linear relationship was observed between the concentration of the extract and the percentage of inhibition of bacterial growth. The higher the concentration of the extract, the greater the percentage reduction in bacterial growth; this is consistent with the Pearson correlation coefficient (0.84 < r < 0.98) for all isolates. Furthermore, the hypothesis test on the correlation coefficient yielded a *p*-value < 0.05, which indicates that, with 95% confidence, there was a significant linear relationship.

**Figure 3:**
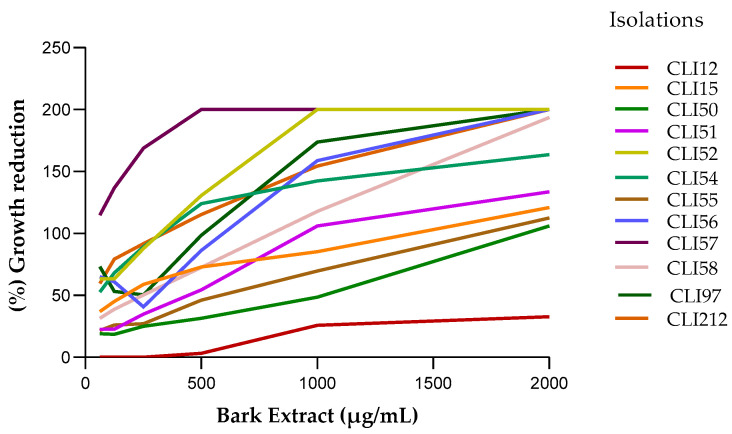
Reduction in growth of *P. aeruginosa* isolates exposed to the ethanolic bark extract. A positive linear relationship was observed between the concentration of the extract and the percentage of inhibition of bacterial growth.

The ethanolic extracts from the wood and bark of *D. vallicola* showed antibacterial activity against all of the clinical isolates of *P. aeruginosa* studied; we observed a reduction in the percentage of bacterial growth treated with the extracts compared to the untreated isolates used as a control. Figure 2 and Figure 3 show a similar tendency among isolates to increase the percentage of growth reduction as the concentration of wood and bark extracts increased, respectively. The wood extract, at the lowest concentration evaluated (62.5 μg/mL), achieved more than 50% growth inhibition of the CLI15, CLI50, and CLI52 isolates. With the bark extract (62.5 μg/mL), more than 50% and 100% growth reduction were achieved in the isolates CLI52, CLI54, CLI56, CLI57, CLI97, and CLI212. 

Table 1 shows the MIC_90_ values of the extracts for each of the clinical isolates evaluated. The effect of the extracts on the inhibition of *P. aeruginosa* varied between each isolate. The lowest MIC_90_ values of the wood (180 μg/mL) and bark (62.5 μg/mL) extracts were observed for the isolates CLI15 and CLI57, respectively, while the highest MIC_90_ values of the wood (3800 μg/mL) and bark (6300 μg/mL) extracts were observed for the CLI12 isolate.

### 2.3. Biofilm Reduction

**Figure 4:**
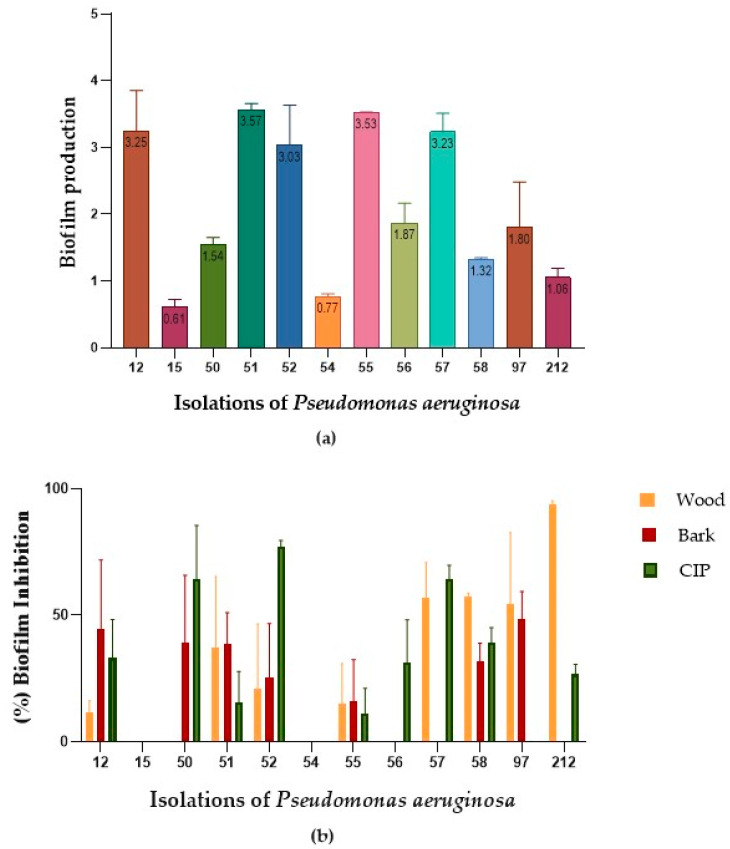
Effect of the bark and wood extracts on *P. aeruginosa*. (**a**) Biofilm formation at 37 °C for 24 h. (**b**) Percentage reduction of mature biofilms after 1 h of treatment with the MIC_90_ of each extract and CIP (2 μg/mL).

All *P. aeruginosa* isolates produced biofilms of different proportions. The isolates CLI12, CLI51, CLI52, CLI55, and CLI57 were strong producers of biofilms in polystyrene microplates, while the rest of the isolates were weak and moderate producers of biofilms (Figure 4a). The effect of the bark and wood extracts against *P. aeruginosa* biofilms varied between each isolate. Table 2 shows the inhibition percentages when the MIC_90_ of the extracts was added to the biofilms formed in each isolate; a percentage of biofilm biomass reduction between 11.5% and 93.7% was obtained for the wood extract and between 16.0% and 48.4% for the bark extract (Figure 4b) after 1 h of exposure. Meanwhile, the percentage of biofilm biomass reduction in cells treated with ciprofloxacin (CIP) ranged between 0.00% and 77.19%. The Kruskal–Wallis test, with a *p*-value of <0.64, and the Dunnett test (0.99 ˃ *p* < 0.70) for the wood and bark extracts, respectively, indicated that there were no statistically significant differences between the effect of CIP and the evaluated extracts in inhibiting mature biofilms in *P. aeruginosa*.

### 2.4. Effect of the Extracts on Cell Membrane Integrity

#### 2.4.1. Leakage of Nucleic Acids and Proteins through the Cell Membrane

**Figure 5:**
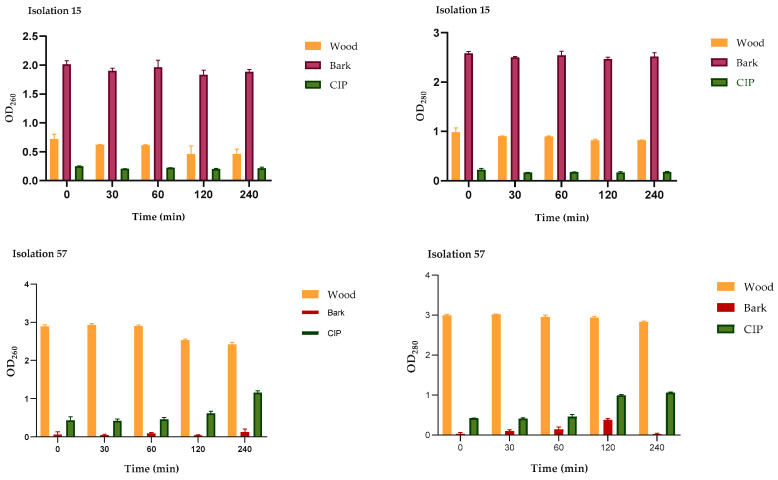
Release of the intracellular content at 260/280 nm versus the time of *P. aeruginosa* treated with the wood and bark extracts (MIC μg/mL) and CIP (2 μg/mL). The figure shows the OD_260_/OD_280_ values of the CLI15 and CLI57 isolates treated with the extracts and CIP at different times. The results are expressed as the absorbance of the sample (treated with extracts) minus the absorbance of the control (samples without extracts).

The action of the wood and bark extracts on the integrity of *P. aeruginosa* membranes was evaluated by release assays of intracellular constituents that absorb at 260/280 nm, such as nucleic acids and proteins. These tests were carried out at 0, 30, 60, 120, and 240 min after treatment with the MIC of the extracts for each isolate. As seen in Figure 5, the effect of the extracts on the integrity of the cell membrane varied between the isolates, but in all cases, leakage of the intracellular material was observed. In the CLI15 isolate, the OD_260_/OD_280_ values in the groups treated with the extracts were significantly higher from time zero compared to the groups treated with CIP. A greater effect of the bark extract was highlighted, while for the CLI57 isolate, a greater effect was observed for the wood extract. These results suggest an alteration in the integrity of the cell membrane of *P. aeruginosa* caused by the extracts.

#### 2.4.2. Measurement of the Extracellular pH

**Figure 6:**
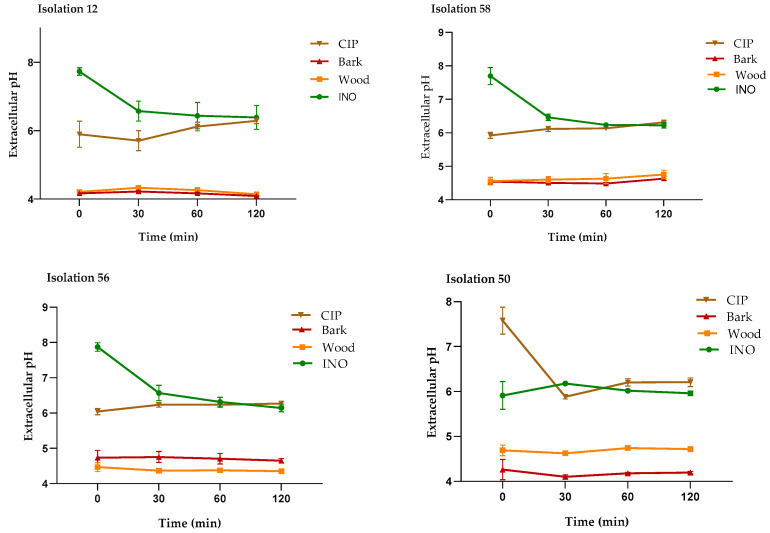
Measurement of the extracellular pH of *P. aeruginosa* treated with *D. vallicola* extracts, untreated cells (INO), and CIP (2 μg/mL). The results of the ANOVA indicate that there were significant differences between the extracts compared to the INO control (*p* < 0.0001) for all isolates. In the cells treated with CIP, there were no significant differences with respect to those cells without treatment (control). The Dunnett test, with a confidence level of 95%, indicates that there were significant differences between the effect of the extracts on the extracellular pH values compared to the effect of CIP.

The extracellular pH measurements of *P. aeruginosa* treated with the extracts, CIP, and untreated cells are shown in Figure 6. As seen in the four isolates, the cells treated with the MIC of the bark and wood extracts showed a significant decrease in extracellular pH compared to the untreated cells (INO) and cells treated with CIP (2 µg/mL); this behavior was similar in all *P. aeruginosa* isolates. These results support the alteration in the integrity of the cell membrane caused by treatment with *D. vallicola* extracts.

#### 2.4.3. LIVE/DEAD Assays

**Figure 7:**
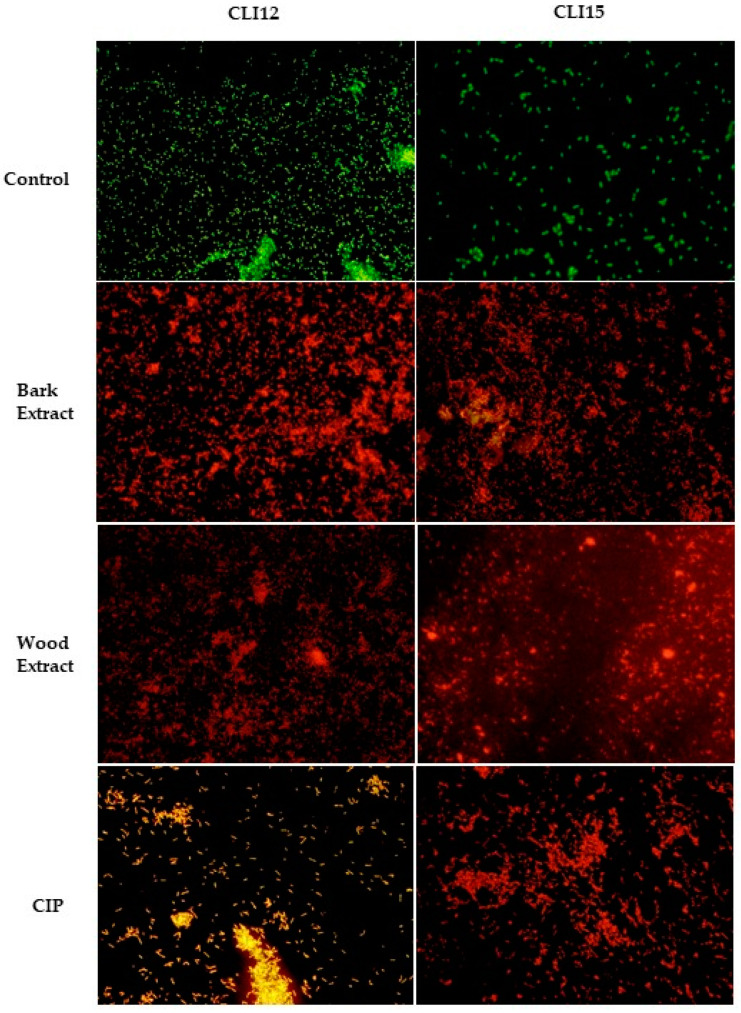
Fluorescence microscopy of *P. aeruginosa* without treatment (control), treated with the bark and wood extracts and CIP after 24 h. Live cells with intact membranes appear green, while dead cells with damaged membranes appear red and orange.

Isolates of *P. aeruginosa* treated with the extracts of *D. vallicola*, CIP and without treatment were observed under fluorescence microscopy. Acridine orange (AO) diffuses through intact cytoplasmic membranes in living cells, where it interacts with DNA, emitting bright green fluorescence. In contrast, ethidium bromide (EB) only penetrates cells with damaged membranes and cell walls in dead cells, intercalates with DNA, and emits orange-red fluorescence [35]. As seen in Figure 7, untreated cells (control), exhibiting completely green fluorescence, grew well after 24 h, while dead cells, characterized by red fluorescence, were prominently observed in the groups treated with the extracts. Similar results were noted in the group treated with CIP.

## 3. Discussion

*Pseudomonas aeruginosa* is a common cause of healthcare-associated infections, particularly pneumonia, and it presents in patients with structural lung diseases such as cystic fibrosis. The increasing trend of resistance to antimicrobials (including multidrug-resistant (MDR) isolates) in recent years, as well as the presence of various virulence mechanisms that increase their ability to cause serious infections [10,36,37,38], represent a significant challenge in the treatment of this pathogen. This piqued our interest in searching for compounds and new therapeutic options for controlling *P. aeruginosa*, with products of plant origin presenting an excellent alternative.

In this research, we reported the antibacterial activity of ethanolic extracts from the bark and wood of *D. vallicola* against intrahospital isolates of *P. aeruginosa* for the first time. The effect of the extracts was different between the isolates and, in all cases, it was concentration-dependent. These results align with those reported by Sousa [39], who found differences in the efficiency of essential oils extracted from the bark of *D. lanceolata* against the strains *Staphylococcus aureus*, *Streptococcus pyogenes*, *Escherichia coli*, *P. aeruginosa*, and *Candida albicans* since the antimicrobial effect varies depending on the concentration and the microorganism evaluated. Studies carried out with extracts from other species of the genus—*D. gardneriana*, *D. moricandiana*, and *D. quitensis*—have also reported antibacterial activity against *S. aureus*, *S. pyogenes*, and *E. coli*, attributing this effect to the content of terpenes present in the essential oils evaluated [40].

Extracts of *D. vallicola* have rarely been studied; the presence of various types of isoquinoline alkaloids has been demonstrated, including pseudopalmatine, isoboldine, isocorydine, N-methylaurotetanine, oliveridine, and oliveroline [22]; however, studies on the antimicrobial activity of its extracts or compounds have not been reported. Alkaloids are a large and structurally diverse group of compounds that serve as scaffolds for important antibacterial drugs such as metronidazole and quinolones. They have been widely studied, not only for their antibacterial potential but also as antibiotic enhancers; furthermore, the effects of these compounds on virulence gene regulatory systems, such as quorum sensing and virulence factors, have also been reported [41,42,43]. Isoquinoline alkaloids of plant origin comprise a broad source of multimodal agents with unique structural diversity and a varied range of pharmacological activities [44]. They are of great interest due to their promising biological activity [45], highlighting their antibacterial, antifungal, antiviral, and antiparasitic activities, among others [46]. Likewise, previous studies [47,48] have documented the antibacterial structure–activity relationship of isoquinoline alkaloids of the protoberberine, protopine, benzophenanthridine, aporphine, and bisbenzylisoquinoline types, providing evidence of damage to membrane and cell wall integrity, inhibition of efflux pumps and related enzymes, as well as damage to bacterial DNA and protein synthesis, as mechanisms of antibacterial action of these compounds. Taking into account the great antibacterial potential reported for isoquinoline alkaloids and the presence of a diverse group of these alkaloids in *D. vallicola*, we could suggest their contribution to the inhibitory activity of ethanolic extracts of wood and bark against *P. aeruginosa*. 

The notorious persistence of *P. aeruginosa* in clinical environments is attributed to its ability to form antibiotic-resistant biofilms; these function as a framework to enclose bacteria on surfaces and protect them from environmental stress, preventing phagocytosis and, therefore, affording them the capacity for colonization and persistence and contributing to their multidrug resistance [49,50,51]. We reported the ability of ethanolic extracts of bark and wood to inhibit mature biofilms in *P. aeruginosa* (48.4% to 93.7%), this effect being similar to that observed in cells treated with CIP in some cases. The antibiofilm activity of species of the genus *Duguetia* had not been reported before.

Inhibition of biofilm formation by berberine in *P. aeruginosa* [52] and *S. epidermidis* [53] has been reported. Likewise, the action of sanguinarine, a benzophenanthridine alkaloid of plant origin, in inhibiting bacterial biofilms has been documented [54], and the action against fungal biofilms of the chelerythrine–sanguinarine combination has also been demonstrated [55]. Taking into account the content of isoquinolinic alkaloids present in *D. vallicola* extracts [22], these could, at least in part, be responsible for the antibiofilm action evidenced in this research. These results represent the first report of the antibiofilm action of *D. vallicola* extracts against *P. aeruginosa*. 

On the contrary, the experiments measuring the leakage of intracellular material (260/280), extracellular pH, and fluorescent staining with AO and EB showed alterations in the integrity of the cell membrane. This indicates that the ethanolic extracts of *D. vallicola* altered the integrity of the membrane of these pathogens as an antibacterial mechanism, leading to cell death. Our results provide new and important insights into the antibacterial and antibiofilm potential of *D. vallicola* extracts against *P. aeruginosa*. Furthermore, we demonstrated the cell membrane as a possible target of its action; however, more studies are required to elucidate the mechanisms involved in the antimicrobial activity of this plant species. This research provides a reference for future research on the mechanisms of antimicrobial action against pathogenic bacteria of intrahospital origin.

## 4. Materials and Methods

### 4.1. Reagents

Mueller–Hinton broth (MHB) (Sigma, Mendota Heights, MN, USA) was used for the determination of the MIC and cultures of bacterial isolates. Tryptic Soy Agar (TSA) and Tryptic Soy Broth (TSB) (Becton, Dickinson and Company, San Diego, CA, USA), Mueller–Hinton agar (MHA) (Sigma, Mendota Heights, MN, USA), and Brain Heart Infusion (BHI) broth (Sigma-Aldrich, St. Louis, MO, USA) were also used for the bacterial cultures. The dimethyl sulfoxide (DMSO), phosphate-buffered saline (PBS), crystal violet (CV), acridine orange (AO), ethidium bromide (EB), and antibiotic ciprofloxacin (CIP) used in this study were obtained from Sigma-Aldrich, St. Louis, MO, USA. Meanwhile, the ammonium hydroxide (NH_4_OH), dichloromethane (DCM), hydrochloric acid (HCl), methanol (MeOH), and glacial acetic acid were obtained from Carlo Erba Reagents, Milan, Italy. 

### 4.2. Obtaining the Extracts

The wood and bark of *Duguetia vallicola* were collected from a specimen located in the El Corozo village (8°33′39″ N, 75°45′30″ W) municipality of Montería Córdoba, a specimen rests in the Herbarium of the Botanical Garden Joaquín Antonio Uribe, from the city of Medellín under collection number JAUM 037841. The plant material, free of impurities, was dried at room temperature and pulverized in a knife mill. The dried and ground material (250 g of bark and 300 g of wood) was extracted separately by percolation with 96% ethanol until exhaustion. Each extract was concentrated in a rotary evaporator (Hei-VAP Core, Wood Dale, IL, USA) until 32 g (12.8% yield) of ethanolic bark extract and 11.4 g (3.8% yield) of ethanolic wood extract were obtained.

### 4.3. Obtaining Alkaloids from the Wood Extract

An 8 g fraction of the ethanolic wood extract was alkalinized to pH 8–9 with 100 mL of 5% ammonium hydroxide (NH_4_OH) and extracted twice with 100 mL portions of dichloromethane (DCM). The organic phase was partitioned with 150 mL of 3% hydrochloric acid (HCl), and the aqueous phase obtained was alkalinized with NH_4_OH until pH 8–9. Finally, it was extracted twice with 100 mL portions of DCM, obtaining a total alkaloid fraction of 235 mg (0.078% of the dry weight of the wood). The fractionation and purification of the total alkaloids were carried out by column chromatography (CC) using silica gel 60 (0.063–0.200 mm), Merck^®^ (De Soto, KS, USA), eluted with DCM/MeOH mixtures ranging from 99:1 to 90:10, and preparative thin-layer chromatography (PTLC) plates, silica gel 60 F_254_, 1 mm, Merck^®^. Chromatographic monitoring was carried out with aluminum thin-layer chromatography (TLC) plates and silica gel coated with fluorescent indicator F_254_ Merck^®^. Initially, 200 mg of total alkaloids were subjected to CC in silica gel (25 g) eluted with DCM/MeOH 99:1, collecting 9 fractions of 10 mL each. Fraction 2 (89 mg) was subjected to CC on silica gel (15 g) to obtain 11.4 mg of liriodenine (1). Fraction 3 (48 mg) was subjected to PTLC using the DCM/MeOH 97:3 elution system, obtaining 16.1 mg of O-methylmoschatoline (2). Fraction 6 (35 mg) was subjected to PTLC using the DCM/MeOH 95:5 elution system, obtaining 7.8 mg of nornuciferin (3). Their structures were established by comparison of their physical and spectral data with those published in the literature, using ^1^H-NMR, ^13^C-NMR, DEPT, COSY ^1^H-^1^H, HMQC, and HMBC spectra, performed on a 400 MHz Bruker Advance DRX spectrometer, in deuterated chloroform (CDCl_3_). The mass spectrum was obtained in electron impact ionization mode at 70 eV.

### 4.4. Strains

Twelve clinical isolates of *P. aeruginosa* (CLI12, CLI15, CLI50, CLI51, CLI52, CLI54, CLI55, CLI56, CLI57, CLI58, CLI97, and CLI212) were used in this study. The isolates were cultured from the blood and urine culture samples of patients hospitalized at the Social Health Service S.A.S. in the city of Sincelejo, Colombia. All microorganisms were identified using standard methods: Vitek^®^ 2 Compact. Biomerieux SA (Marcy-l’Étoile, France). BHI medium and cetrimide agar were used to maintain the cultures until testing was performed.

### 4.5. Antibacterial Susceptibility Testing

The minimum inhibitory concentration (MIC) of the ethanolic extracts from the wood and bark of *D. vallicola* against the clinical isolates was defined as the lowest concentration at which 90% (MIC_90_) of bacterial growth was inhibited, compared to the control (untreated cells). The MIC_90_ was determined by performing broth microdilution assays using 96-well microtiter plates (Nunclon Delta, Thermo Fisher Scientific, Waltham, MA, USA), as described in the Clinical Laboratory Standards Institute method M07-A9 (CLSI) [56], with minor modifications. Serial dilutions were performed in MHB to obtain final concentrations of 2000, 1000, 500, 250, 125, and 62.5 μg/mL of the extracts in each reaction well. To carry out the experiments, a stock solution of each extract was prepared at 25,000 μg/mL in 10% DMSO. Cells without treatment and cells treated with 2 μg/mL of CIP were used as negative and positive controls, respectively. The assays were developed at a final volume of 200 µL per well as follows: 100 µL of bacterial inoculum at a concentration of 10^8^ CFU/mL and 100 µL of the extracts, adjusted to reach the previously described concentrations in the final reaction wells. Wells with bacterial inoculum, without extracts, and with CIP (2 μg/mL) were used as growth and positive controls, respectively. The plates were incubated at 37 °C for 24 h. The experiments were performed in triplicate. The inhibition of bacterial growth by the extracts was determined by changes in optical density using a SYNERGY LX microplate reader (Biotek, Winooski, VT, USA) at 600 nm from the beginning of incubation to the end of incubation (24 h). Finally, the percentage of inhibition of bacterial growth [57] was calculated using the following equation:

%Inhibition = (1 − (OD_t24_ − OD_t0_/OD_gc24_ − OD_gc0_)) × 100

where OD_t24_ is the optical density of the test well at 24 h post-inoculation; OD_t0_ is the optical density of the test well at 0 h post-inoculation; OD_gc24_ is the optical density of the growth control well at 24 h post-inoculation; OD_gc0_ is the optical density of the growth control well at 0 h post-inoculation.

### 4.6. Quantitative Assessment of Biofilm Formation

The effect of *D. vallicola* wood and bark extracts on mature *P. aeruginosa* biofilms was evaluated following the protocol described by [58,59], with minor modifications. For biofilm formation, bacterial colonies from 24 h of incubation in TSA were used, standardizing the bacterial inoculum to 10^8^ cells/mL. Then, in 96-well polystyrene microplates, 200 µL of the bacterial inoculum was discharged into each well and incubated at 37 °C for 24 h. Subsequently, the broth was removed from the microplates, and 200 µL of the wood and bark extracts (evaluated separately), at the MIC concentration of each isolate, was added to the TSB broth and incubated at 37 °C for 1 h. Subsequently, the floating cells were removed, and the biofilms from the bottom of the wells were washed with deionized water. Excess moisture was then removed by tapping the microplates on sterile napkins, and the plates were dried for 5 min. Six replicates of each experiment were performed. Cultures without extracts were used as a negative control, and cultures with CIP were used as a positive control. Biofilm reductions were quantified by staining the wells with 200 µL of 0.1% CV for 20 min. The samples were washed with deionized water until the excess dye was removed; the excess water was carefully dried, and then the CV was solubilized in 250 µL of 30% glacial acetic acid. Absorbance values were measured at 590 nm (OD_590_) using a SYNERGY LX microplate reader (Biotek). Biofilm production was grouped into the following categories: OD_590_ < 0.1, non-producers (NPs); OD_590_ 0.1–1.0, weak producers (WPs); OD_590_ 1.1–3.0, moderate producers (MPs); OD_590_ > 3.0, strong producers (SPs). Biofilm reduction was calculated [60] using the following equation:

% Biofilm reduction: AbsCO − AbsExt/AbsCO × 100

where AbsCO is the absorbance of the control sample, and AbsExt is the absorbance of the sample treated with the extracts.

### 4.7. Effect of the Extracts on Cell Membrane Integrity

Evaluation of the action of the ethanolic extracts from the wood and bark of *D. vallicola* on the cell membrane of *P. aeruginosa* was carried out through experiments of intracellular material leakage, measurement of extracellular pH, and cell fluorescence microscopy treated with each of the evaluated extracts.

#### 4.7.1. Leakage of Nucleic Acids and Proteins through the Cell Membrane

The release of intracellular material was measured according to the methodology proposed by [61], with some modifications. Cells cultured in nutrient broth were centrifuged at 3000 g for 20 min, washed three times, and resuspended in 20 mL of PBS (pH 7.0). The cell suspension was then treated with the wood and bark extracts (MIC for each isolate; separate experiments) and incubated at 37 °C for 0, 30, 60, and 120 min. Subsequently, 2 mL of the samples was collected and centrifuged at 3000 g for 20 min. Then, to determine the concentration of the released constituents, 2 mL of the supernatant was used to measure the absorbance at 260/280 nm with a Spectroquant^®^ Prove 300 UV/Vis spectrophotometer (Merck KGaA, Darmstadt, Germany). Samples without extracts and samples with CIP were used as controls. All assays were performed in triplicate.

#### 4.7.2. Measurement of Extracellular pH

Measurement of the extracellular pH of *P. aeruginosa* after treatment with *D. vallicola* wood and bark extracts was determined according to [60], with some modifications. First, 20 mL of the bacterial suspension (1 × 10^7^ CFU/mL) in nutrient broth was incubated at 37 °C for 24 h. The samples were then centrifuged at 3000 g for 20 min; then, the sediment was collected, resuspended, and washed three times with double-distilled water, followed by resuspension again in 20 mL of sterile double-distilled water. After the addition of the extracts (MIC of each isolate), the extracellular pH of *P. aeruginosa* was determined at 0, 30, 60, and 120 min using a Schott^®^ Instruments (Mainz, Germany) Handylab pH 11 pH meter. They were used as control samples without extracts and samples with CIP.

#### 4.7.3. LIVE/DEAD Assays

The LIVE/DEAD assays were developed following the methodology proposed in [35]. A suspension of *P. aeruginosa* (10^7^ CFU/mL) was placed on sterile slides and incubated for 24 h. The cells were then washed three times with PBS. Subsequently, the MIC of the extracts of each isolate and CIP were added to the experimental groups, and the bacterial inoculum in nutrient broth was used as a control. The prepared slides were incubated at 37 °C for 24 h and then washed three times with PBS. Together, AO (5 µL, 100 mg/L) and EB (5 µL, 100 mg/L) were mixed under dark conditions and added to the slides under dark conditions for 30 s. The samples were then observed on an Olympus BX43 fluorescence microscope (Tokyo, Japan) and photographed with a DP72 camera.

### 4.8. Data Analysis

The results were analyzed using GraphPad Prism version 8.0 software and Microsoft Excel version 2404. Initially, the Shapiro–Wilk test was used to determine the distribution of the data. Subsequently, Pearson’s correlation coefficients were used to measure the degree of linearity, the correlation between the concentration of the extracts, and the percentage reduction in bacterial growth. To compare the effects of the extracts and CIP on biofilm reduction, the Kruskal–Wallis and Dunnett tests were used; both tests was also used to compare the effects of the extracts and CIP on the leakage of intracellular material through the membrane (260/280 nm) and to compare the effects of the treatments on the extracellular pH of *P. aeruginosa*.

## 5. Conclusions

In this study, we investigated the antibacterial potential of ethanolic extracts of *Duguetia vallicola* wood and bark against clinical isolates of *Pseudomonas aeruginosa*, as well as their role in biofilm inhibition. Furthermore, we explored the action against the cell membranes of these pathogens. We demonstrated the antibacterial action of the extracts under study against *P. aeruginosa*, with this effect being associated with damage to the integrity of the cell membrane, in addition to its action against bacterial biofilms. Additionally, we reported the presence of three isoquinoline alkaloids in the wood extract of *D. vallicola*. It is necessary to continue these studies, with the aim of elucidating the mechanisms of antibacterial action and the composition of the extracts of this species, which hold promise as an alternative tool for the treatment and control of intrahospital pathogens such as *P. aeruginosa*.

## Figures and Tables

**Table 1:** Minimum inhibitory concentration (MIC_90_) values of the ethanolic extracts of wood and bark (μg/mL) against clinical isolates of *P. aeruginosa*.

*P. aeruginosa*	Wood Extract	Bark Extract
	MIC_90_	MIC_90_
CLI 12	3800	6300
CLI 15	180	1460
CLI 50	500	2200
CLI 51	3000	1600
CLI 52	490	73
CLI 54	1270	300
CLI 55	3120	2000
CLI 56	1480	392
CLI 57	2600	62.5
CLI 58	2790	794
CLI 97	1560	250
CLI 212	1430	100

**Table 2:** Percentages of inhibition of mature biofilms of the ethanolic extracts of wood and bark vs. CIP in *P. aeruginosa*.

*P. aeruginosa*	Wood	Bark	CIP
CLI12	11.51	44.79	33.03
CLI15	0.00	0.00	0.00
CLI50	0.00	38.97	64.09
CLI51	37.09	38.85	15.49
CLI52	20.91	25.51	77.19
CLI54	0.00	0.00	0.00
CLI55	15.20	16.07	11.28
CLI56	0.00	0.00	31.41
CLI57	56.79	0.00	64.19
CLI58	57.58	31.80	39.07
CLI97	54.31	48.46	0.00
CLI212	93.74	0.00	26.63

## Data Availability

The data presented in this study are available in the article.

## Supplementary Materials

The following supporting information can be downloaded at https://www.mdpi.com/article/10.3390/plants13101412/s1, Table S1. NMR spectroscopic data (^1^H 400 MHz, ^13^C 100 MHz) of **1** in CDCl_3_. Table S2. NMR spectroscopic data (^1^H 400 MHz, ^13^C 100 MHz) of **2** in CDCl_3_. Table S3. NMR spectroscopic data (^1^H 400 MHz, ^13^C 100 MHz) of **3** in CDCl_3_. Figure S1. ^1^H-NMR spectrum (amplification 6.2–9.1 ppm) of **1** (CDCl_3_, 400 MHz). Figure S2. ^1^H-NMR spectrum (amplification 4.0–9.2 ppm) of **2** (CDCl_3_, 400 MHz). Figure S3. ^1^H-NMR spectrum (amplification 7.5–9.2 ppm) of **2** (CDCl_3_, 400 MHz). Figure S4. ^1^H-NMR spectrum of **3** (CDCl_3_, 400 MHz). Figure S5. ^1^H-NMR spectrum (amplifications) of **3** (CDCl_3_, 400 MHz).
