# Supplementary material for: Antibacterial and Antibiofilm Potential of Ethanolic Extracts of Duguetia vallicola (Annonaceae) against in-Hospital Isolates of Pseudomonas aeruginosa
Source: Plants (Basel). 2024 May 18;13(10):1412. doi: 10.3390/plants13101412 (PMC11126144; doi:10.3390/plants13101412)
Supplement: Supplementary file 1 [file plants-13-01412-s001.zip › plants-2952526-supplementary.pdf]

plants

Article

Antibacterial and antibiofilm potential of ethanolic extracts of *Duguetia vallicola* (Annonaceae) against in-hospital isolates of *Pseudomonas aeruginosa*

Orfa Inés Contreras-Martínez[1], Daniela Sierra-Quiroz[1] and Alberto Angulo-Ortíz[2*]

[1] Biology Department, Faculty of Basic Sciences, University of Córdoba, Montería 230002, Colombia
[2] Chemistry Department, Faculty of Basic Sciences, University of Córdoba, Montería 230002, Colombia
* Correspondence: aaangulo@correo.unicordoba.edu.co

Supplementary Materials

Table S1. *NMR Spectroscopic Data (^{1}H 400 MHz, ^{13}C 100 MHz) of* **1** *in* $CDCl_3$

ATOM	^{1}H (ppm)	MULT	*J* (Hz)	^{13}C (ppm)	DEPT	HMQC	HMBC
1				147.9	C		7.15, 6.36
1a				130.2	C		
1b				123.2	C		
2				151.7	C		7.15, 6.36
3	7.15	s		103.2	CH	7.15	7.74
3a				135.7	C		8.88
4	7.74	d	5.0	124.2	CH	7.74	8.88
5	8.88	d	5.1	144.8	CH	8.88	
6a				145.2	C		8.88
7				182.3	C		8.56
7a				131.2	C		7.55
8	8.56	dd	7.9 – 0.7	128.8	CH	8.56	7.71
9	7.55	pst	7.7 – 7.4	128.5	CH	7.55	8.59
10	7.71	dt	8.2 – 1.2	133.9	CH	7.71	
11	8.59	d	8.1	127.3	CH	8.59	7.55
11a				132.8	C		8.59, 7.71
12	6.36	s		102.4	CH_2	6.36	6.36

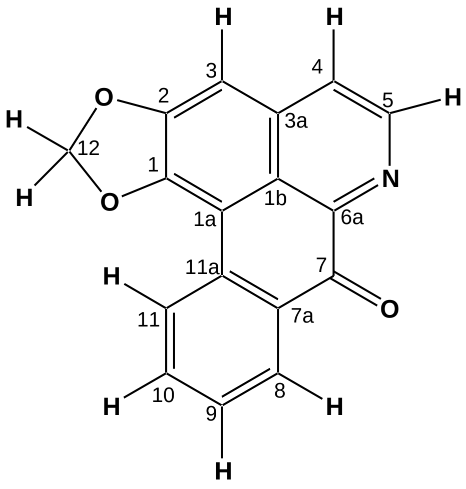

1

Table S2. *NMR Spectroscopic Data (^{1}H 400 MHz, ^{13}C 100 MHz) of* **2** *in $CDCl_3$*

ATOM	^{1}H (ppm)	MULT	*J* (Hz)	^{13}C (ppm)*	HMQC	HMBC
1				156.4		4.08
1a				115.8		9.11
1b				122.8		8.22
2				147.2		4.10
3				148.4		8.22, 4.19
3a				130.8		8.97
4	8.22	d	5.3	119.1	8.22	8.97
5	8.97	d	5.3	144.6	8.97	
6a				145.6		8.97
7				182.6		8.58
7a				131.1		9.11, 7.54
8	8.58	dd	7.8 – 1.4	129.0	8.58	7.75
9	7.54	dt	7.9 – 1.0	128.2	7.54	9.11
10	7.75	dt	8.5 – 1.6	134.3	7.75	8.58, 7.75
11	9.11	d	8.3	127.5	9.11	7.54
11a				131.5		9.11, 7.54
12	4.08	s		61.0	4.08	
13	4.10	s		61.4	4.10	
14	4.19	s		61.7	4.19	

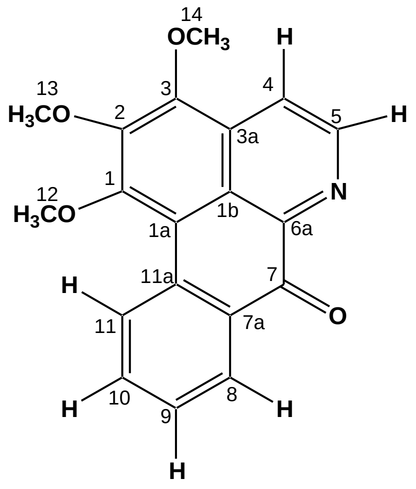

2

Table S3. *NMR Spectroscopic Data (^1H 400 MHz, ^{13}C 100 MHz) of* ***3*** *in $CDCl_3$*

ATOM	^1H (ppm)	MULT	*J* (Hz)	^{13}C (ppm)	DEPT	HMQC	HMBC
1				145.7	C		6.65, 3.66
1a				126.8	C		6.65
1b				125.1	C		6.65, 3.12, 3.03
2				152.9	C		6.65, 3.89
3	6.65	s		111.6	CH	6.65	
3a				127.3	C		3.61
4	2.82<br>3.29	dd<br>m	3.2 – 16.4<br>5.6	27.2	CH_2	2.82<br>3.29	6.65, 3.61
5	3.12<br>3.61	m<br>dd	4.2 – 13.8<br>5.9 – 12.1	42.1	CH_2	3.12<br>3.61	
6	4.81	s *broad*					
6a	4.05	m		53.2	CH	4.05	
7	3.03<br>3.12	pst<br>m	13.7<br>4.2 – 13.8	35.6	CH_2	3.03<br>3.12	
7a				131.7	C		3.12, 3.03
8	7.25	m		127.7	CH	7.25	7.32, 3.12
9	7.25	m		128.0	CH	7.25	7.25
10	7.32	m	8.0 – 2.6	127.4	CH	7.32	
11	8.39	d	7.8	128.5	CH	8.39	8.39
11a				134.6	C		8.39, 3.12, 3.03
12	3.66	s		60.2	CH_3	3.66	
13	3.89	s		55.9	CH_3	3.89	

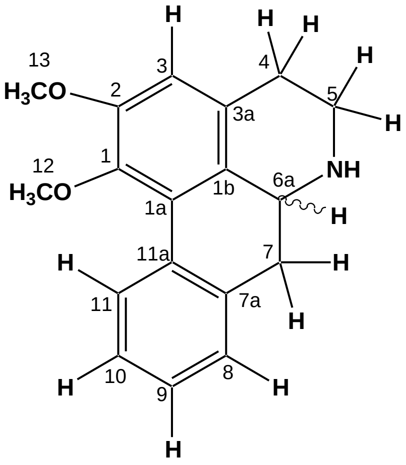

3

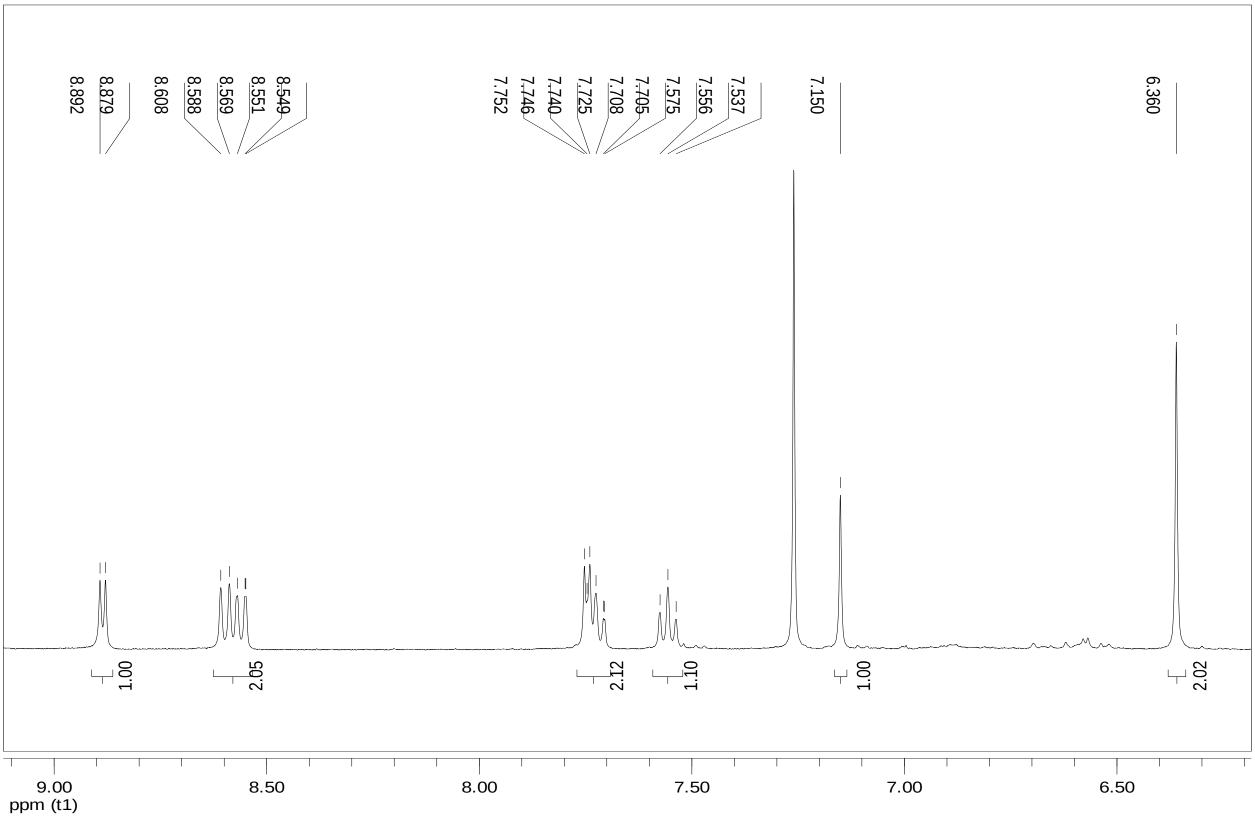

Figure S1. ^{1}H-NMR spectrum (amplification 6.2 – 9.1 ppm) of **1**, ($CDCl_3$, 400 MHz).

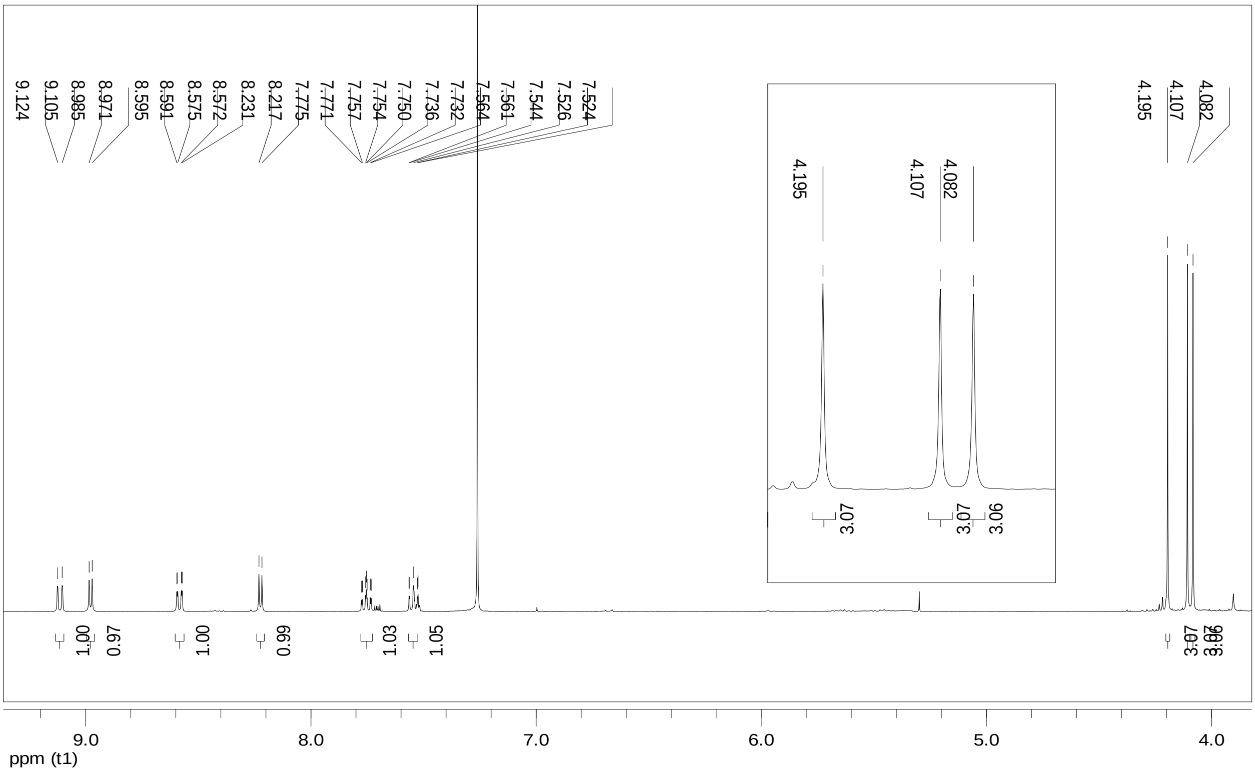

Figure S2. ^{1}H-NMR spectrum (amplification 4.0 – 9.2 ppm) of **2**, ($CDCl_3$, 400 MHz).

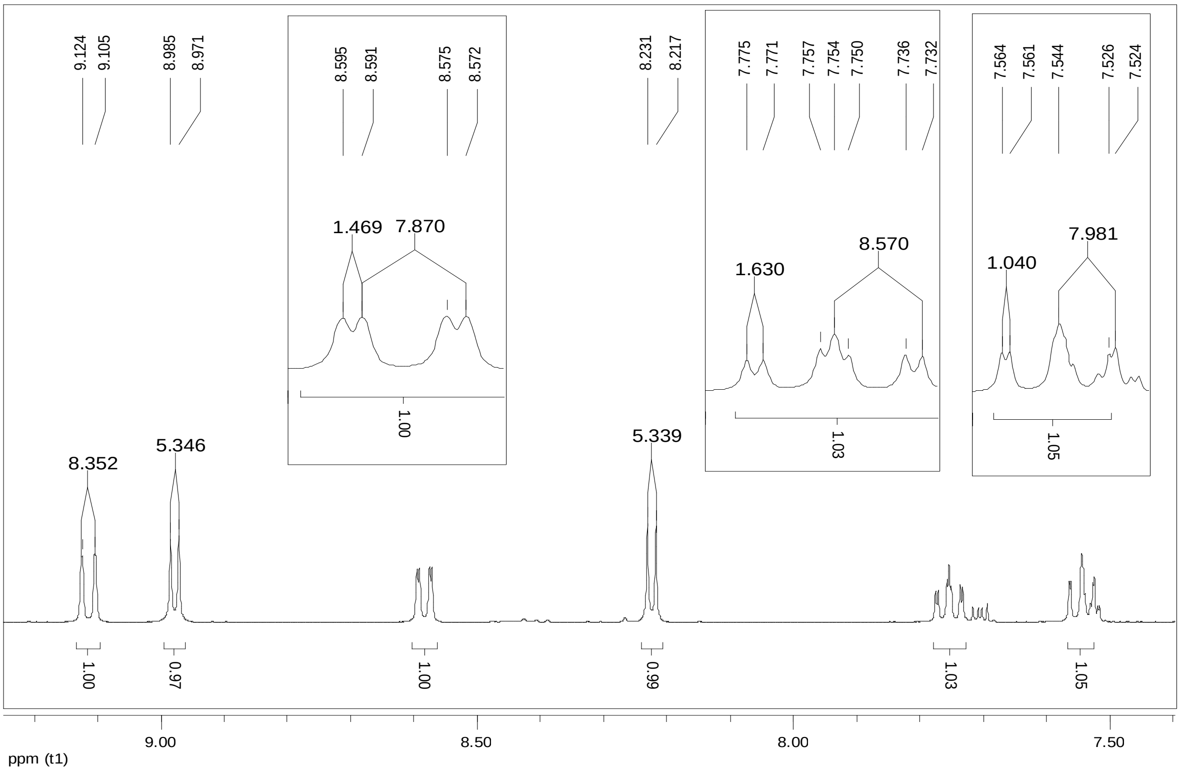

Figure S3. ^{1}H-NMR spectrum (amplification 7.5 – 9.2 ppm) of **2**, ($CDCl_3$, 400 MHz).

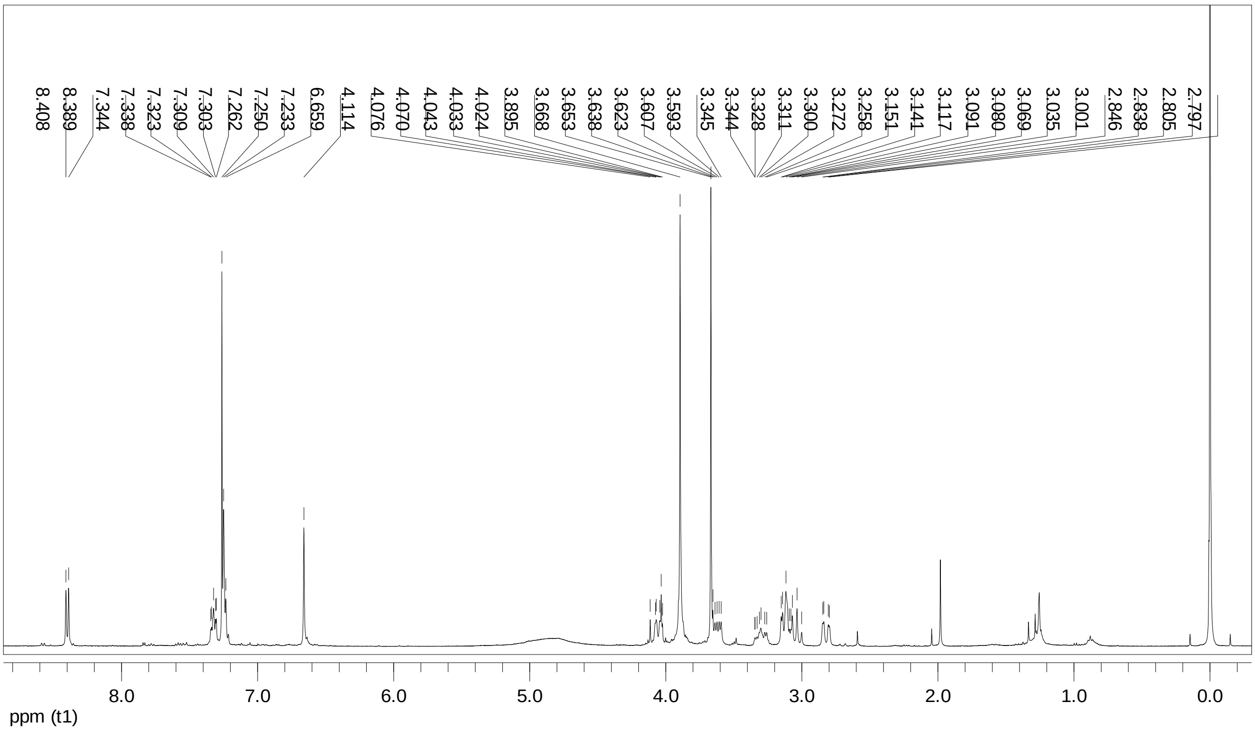

Figure S4. ^{1}H-NMR spectrum of **3**, ($CDCl_3$, 400 MHz).

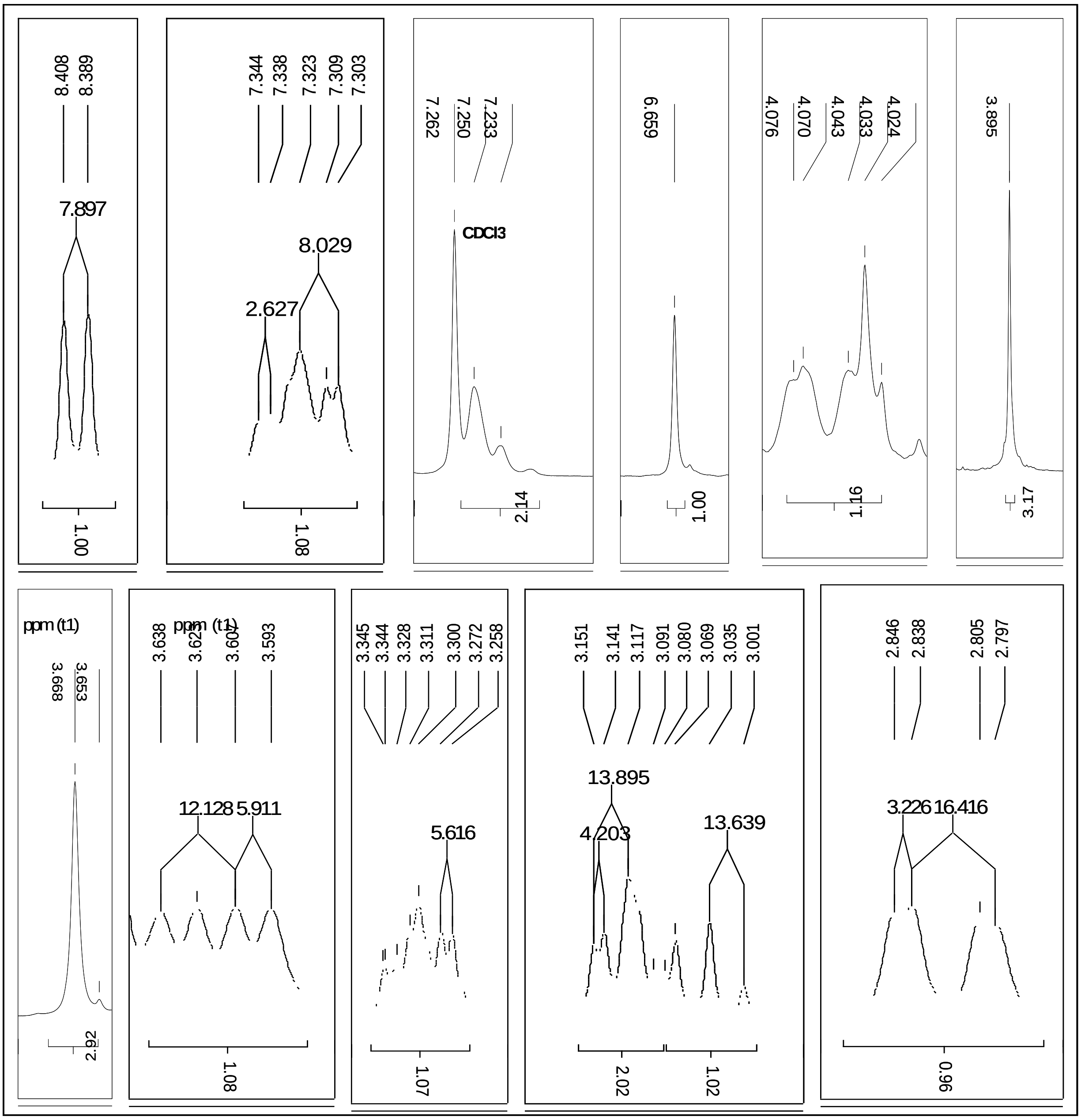

Figure S5. ^{1}H-NMR spectrum (amplifications) of **3**, ($CDCl_3$, 400 MHz).
